# News and Miscellany

**Published:** 1902-03

**Authors:** 


### News and Miscellany.

**The great** and only Lamphear will attend our Dallas meeting, and read a paper.

---

**The next meeting** of the State Board of Medical Examiners will be held in Waco, May 13.

---

**A learned pathologist,** a professor in a medical college, writes, in one of our exchanges, of "a new *epidemic* disease amongst *horses* in the Philippine" Islands.

---

**Another One.**—Dr. Ben F. McQuiston, of Paris, Texas, was shot and killed February 28, ult. A. W. McComas was arrested, charged with the killing. The cause of the killing is understood to be connected with the family of McComas, says the Associated Press dispatch.

**Dr. D. W. Claywell,** of Troy, Texas, died at his home in that city February 14, from carbuncle of the shoulder. He was a member of the East Texas Medical Association.

---

**Dr. A. D. Boggs,** of Marquez, Texas, died of pneumonia at his home February 1, ult. He was a member of the State Association, and of the East Texas Medico-Chirurgical Society.

---

**Dr. G. W. Akard,** of Springtown, Texas, died at his home December 19, 1901. Dr. Akard was a member of the State Medical Association, and had been an assistant surgeon in the Confederate States Army.

---

**A bright little boy,** a friend of mine, [I love boys, bad ones. If I had a "good" boy, I'd whip it out of him.] seeing his mamma's tabby quite sick, said: "Doctor, if I were rich, I'd establish an Infirmity for cats."

---

**On account** of the recent death of my father, Dr. D. W. Claywell, I have for sale a bran new McIntosh sixteen plate electric static machine, with X-ray attachments. Will sell cheap. Apply to J. W. Claywell, State University, Austin, Texas.

---

**I want** to call attention to the Ozark Sanitarium, at Hot Springs, Arkansas, Drs. Jelks and Holland in charge. It is for the treatment of all such cases as seek the baths of the famous Hot Springs, and for surgical and gynecological cases. Dr. Jelks will attend the Dallas reunion of the old Confederate Surgeons.

---

**Be happy** in making others happy. Do all the good you can, as often as ever you can. Live temperate lives in thought, word and deed, and leave all else to God. Above all, don't worry, don't hurry, don't judge,—what's the use?—*Dr. Love in Medical Mirror.*

That's the idea. Take the lean with the fat, and let it go at that.—Ed.

---

**Senator Burrows** says he has a new story, and here it is: "There is an old darkey who has been working for me. He lost his wife—No. 4—the other day, and I was sympathizing with him as best I could, telling him that they would meet in heaven, etc., when the old fellow broke in: 'I know dat, Mars Burrows, I know dat. I ain't makin' no objections. It were de act of a all-wise and unscrupulous Providence.' "—*Ex.*

---

**Convicted of Abortion.**—Dr. Q. C. Smith, of Austin, a well known practitioner of more than twenty years in this city, was indicted by the grand jury for criminal abortion, and was tried at the present session of the Austin district court. The verdict of the jury was "guilty," and the punishment was assessed at two years in the penitentiary. A motion for a new trial was granted, on the ground that at the noon recess one of the jurors had separated himself from the others, and had gone down town to dinner instead of dining with the sheriff. Dr. T. J. Bennett, of Austin, who was called to the woman after Smith had delivered the fetus, was the complainant and prosecuting witness.

---

**Bills, Bills, Bills.** Doctors expect pay at this season. So do editors. Every bill on my books was sent out in November and January, and again in February. If I didn't want and expect the money, I wouldn't do it, for it's lots of trouble. While many, very many, responded, as many or more didn't, and I want to hear from them. I know that it is only negligence—or laziness—that prevents them from remitting. Doctor, you got your bill. "Do it now." Send me a postoffice money order or an express order or a check, or "any old thing" that represents and will fetch a dollar—two—three—five, as the case may be. The north wind doth blow and we shall have snow, and what will poor robin do then—if you don't?

---

**University of Texas Mineral Survey.**—We have received Bulletin No. 2, which deals with the results of the examination of certain public lands in the counties of Brewster, El Paso, Jeff Davis, Pecos, Presidio and Reeves.

It contains two reports on the sulphur deposits of El Paso county, showing that they are well worth attention and that they could supply large quantities of sulphur to commerce.

There is also a description of the deposits of quicksilver ores in Brewster county, showing the extent of the field, the value of the product, and the possibility of further development.

All bulletins, maps, etc., issued by this Survey, are for gratuitous distribution to the citizens of the State, and may be obtained by addressing Wm. B. Phillips, Director of the University of Texas Mineral Survey, Austin, Texas.

---

**The Red-Back** continues to boom. Nearing its seventeenth birthday, it still heads the procession. It is not only the band-wagon, but the band,—the whole push. It is "It." There is nothing like it in heaven or earth nor in the waters beneath the earth; no, not anything whatever. It is indispensable to every doctor who does not wish to get left, and is the best advertising medium extant for things that a doctor ought to have. There is nothing like it. I was offered, (and I accepted the offer), *fifty per cent.* more for a certain preferred page than I had been getting. It is the official organ of the State Association of Health Officers and of most of the district medical societies, and as these are growing, the new subscribers are just pouring in. I'll soon be able to splurge an automobile, and make all the other fellows green with envy, or *shay* green, anyhow, (a wonderful no-horse shay).

---

**The meeting** of the Texas State Medical Association having been changed from El Paso to Dallas, and the date, April 22-25, changed to May 6-9, of course the Committee of Arrangements had to be changed also. Dr. Jno. O. McReynolds, of Dallas, has been appointed chairman of the committee. To him applications should be made for floor space for exhibits, etc. I have received several inquiries on this head. Although the Association invited itself to Dallas, I haven't the slightest doubt but that the Dallas doctors will be glad to see us, a whole lot of us, and will set 'em up. I am reminded here of the resolution adopted by the Brazos Valley Medical Society when accepting Bryan's invitation to hold the next meeting in that city: "Resolved, that the Bryan people do not go to the expense of a banquet. Resolved, that if they do, we'll go to it." Calvert gave us a banquet, (I'm a B. V. man), but the preachers having just previously held forth there, it was a "dry town." The hardest proposition I was ever up against, was having to respond to a toast—a dry one at that—drunk in cold water.

